# Is emotional eating associated with behavioral traits and Mediterranean diet in children? A cross-sectional study

**DOI:** 10.1186/s12889-022-14192-8

**Published:** 2022-09-22

**Authors:** Alessandra Buja, Mariagiovanna Manfredi, Chiara Zampieri, Anil Minnicelli, Roberta Bolda, Filippo Brocadello, Maura Gatti, Tatjana Baldovin, Vincenzo Baldo

**Affiliations:** 1 Hygiene and Public Health Unit, Department of Cardiac, Thoracic, Vascular Sciences and Public Health, University of Padua, Via Loredan, 18, 35131 Padova, Italy; 2 Affidea Poliambulatorio Morgagni, Via Cavazzana, 39/2, 35123 Padova, Italy

**Keywords:** Emotional eating, Mediterranean diet, Behavioral traits, Social determinants, Children

## Abstract

**Background:**

Stress and negative emotions may impact on appetite, inducing some individuals to eat less and others to eat more. This behavior has been implicated in the onset of bodyweight problems and eating disorders in childhood. The aim of our study is to evaluate factors potentially associated with emotional eating in children.

**Methods:**

The present cross-sectional study derives from a survey conducted in 2021 on 8–9 years old children attending 11 primary schools. A questionnaire was administered that contained multiple-choice items relating to the children and their mothers, and touching on all the factors thought to be associated with emotional eating as behavioral traits or adherence to Mediterranean diet. A multivariable logistic regression was performed to test the association.

**Results:**

Emotional undereating was positively associated with emotional symptoms (OR 1.72; 95% CI 1.11–2.67); emotional overeating was positively associated with both emotional symptoms (OR 2.01; 95% CI 1.29–3.13) and hyperactivity (OR 2.80; 95% CI 1.59–4.92), and inversely associated with peer problems (OR 0.50; 95% CI 0.25–0.99). Emotional undereating was also positively associated with the number of siblings (OR 1.50; 95% CI 1.03–2.18), and inversely associated with a good adherence to the Mediterranean diet (OR 0.25; 95% CI 0.08–0.84).

**Conclusions:**

The study found children’s emotional eating associated with both dietary patterns and behavioral traits (in particular emotional symptoms, hyperactivity and peer problems). It could be useful to improve parents’ awareness so that they can anticipate and pay more attention to this issue. Adherence to the Mediterranean diet should also be reinforced, by means of health promotion interventions at school, for example.

## Background

Emotional eating is the tendency to change one’s eating behavior in response to emotions [1]. An Italian survey conducted during the COVID-19 lockdown revealed that almost half of the respondents, aged 18–79 years, declared to have used food as a means of comfort in response to their anxious feelings and to be prone to increasing their food intake to feel better [2]. Stress and negative emotions can affect appetite differently, inducing some individuals to eat more and others to eat less. These behaviors, termed emotional overeating (EOE) and emotional undereating (EUE), have been implicated in the onset of bodyweight problems and eating disorders [3] in childhood. The literature suggests an involvement of EOE and EUE in the etiology of bulimia nervosa, binge eating disorder and obesity [4, 5], all of which are risk factors for numerous chronic diseases (e.g., cardiovascular diseases, musculoskeletal complications, diabetes, and cancer) [6].

Children’s eating behavior is complex and influenced by a variety of interacting factors. Story et al. proposed a conceptual framework for understanding factors that influence adolescent eating behaviors and food choices [7]. The levels of influence are individual (e.g. behavioral traits and gender), social environmental (e.g. parents and peers) [7]. Data from the Gemini twin study showed that both EOE and EUE in early childhood are shaped primarily by the shared family environment, not by genes [3], apparently emerging in the preschool years [1]. Many studies concluded that the home environment and parenting behavior are key factors. Parents have a crucial role in shaping children’s eating as the main providers of food, early models of eating behavior, and through the use of parenting and feeding practices. Notably though, children do not passively absorb and reiterate their parent’s behavior, so some aspects of the origins of emotional eating remain unclear [4].

A number of studies have examined whether emotional eating (EE) is associated with energy and macronutrient intake or particular food choices. One found that EE in adults is associated with the consumption of fast food, salty snacks, sweet high-fat foods, energy-dense foods, and artificially sweetened beverages [8]. These palatable foods provide hedonic pleasure and instant reward, which can distract from the experience of negative emotions. Another study showed that EE is associated with unhealthy dietary patterns in children characterized by fast foods, ice cream, fried foods, French fries, potato chips, cakes and sugar-sweetened sodas [9].

Given the paucity of studies providing an overall picture of the risk factors for emotional eating in children, the present study aimed to disentangle the role of various elements potentially involved, such as characteristics of the individuals, their social environments, and their parents, and the adherence to the Mediterranean diet (MD).

## Methods

### Participants

The present cross-sectional study derives from a survey administered in April–May 2021, as part of an educational intervention called “*Le Buone Abitudini* [Healthy Habits] [10]” running since the academic year 2018/2019 at several primary schools in the province of Padua (north-east Italy). This project promotes the adoption of a varied, healthy, and nutritionally balanced diet during childhood. The province of Padua has 69 state primary schools, and 11 of them agreed to participate in the study with at least one voluntary class. All parents of students attending the classes involved in the evaluation study were asked to give their written informed consent to the participation of the children. Moreover, the children’s mothers were asked to anonymously answer a self-administered ad hoc online questionnaire. Of the 379 questionnaires distributed, 185 (response rate of 49%) were completed and returned to the study authors. Seven questionnaires, answered by someone other than the mother, were rejected to make perspective more consistent. Our study sample included a total of 178 children in their third year (8–9 years old) attending 21 classes.

### Materials

The questionnaire contained multiple-choice items relating to both the children and their mothers, and touched on a number of factors that might be associated with emotional eating - i.e., social sphere and demographics, family setting, behavioral traits - as well as the children’s weight and height.

EUE and EOE were measured using two subscales (with four questions each) of the Children’s Eating Behavior Questionnaire (CEBQ) [11]. A previous study supported the use of the CEBQ as a psychometrically sound tool for assessing children’s eating behaviors [12]. The EOE subscale contained the following statements: “My child eats more when anxious”; “My child eats more when annoyed”; “My child eats more when preoccupied”; “My child eats more when angry”. The statements in the EUE subscale were as follows: “My child eats less when anxious”; “My child eats less when angry”; “My child eats less when upset”; “My child eats less when happy”. The statements are scored from 1 = “never” to 5 = “always”. Tertiles were identified for both EUE and EOE, and the third tertile identifies individuals with the more frequent emotional eating behaviors. The use of tertiles allowed to evidence difference among groups characterized by extreme distribution (first versus third tertiles) of the variable, permitting also to have an equal sample sized for each group.

The questionnaire also investigated: whether the children had siblings (yes or no) and, if so, how many; and the hours of sleep per day, including daytime naps. Children’s behavioral traits were measured on the basis of their mothers’ reports using the Italian version of the Strengths and Difficulties Questionnaire (SDQ) [13]. The SDQ uses a 3-point Likert scale (from 0 = “not true” to 2 = “very true”) and consists of five subscales investigating: Emotional Symptoms, Hyperactivity/Inattention, Peer Relationship Problems, Conduct Problems and Prosocial Behavior. Tertiles were identified for each behavioral trait, with the third tertile identifying individuals showing a given behavioral trait the most. Previous study [14] evaluated the internal consistency reliability of each scale of Italian version of SDQ and showed moderate to good values: α = .84 for Conduct Problems, α = .75 for Emotional Symptoms, α = .86 for Hyperactivity-Inattention, α = .68 for Peer Relationship Problems, α = .83 for Prosocial Behavior, and α = .88 for the Total Difficulties score.

The “adherence to the MD” variable was derived from the Italian version of the KidMed Test [15], classifying scores of 8 or more as good, and scores of 7 or less as poor-to-moderate. The development of the KidMed Test took into consideration aspects sustaining Mediterranean dietary patterns and also those undermining it. A previous study evidenced that KIDMED questionnaire is a reliable instrument for assessing adherence to the Mediterranean diet [16]. The KidMed index ranges from 0 to 12, based on a 16-question test. Questions with a negative connotation vis-à-vis adherence to the MD were assigned a value of − 1, and those with a positive connotation scored + 1. Children were classified as underweight/regular weight or overweight/obese using the International Obesity Task Force cut-offs, as suggested by Cole et al. [17].

The second part of the questionnaire covered socio-economic aspects referring to mothers and family environments, including: the mother’s citizenship (Italian or other); the mother’s education (middle school or less, high-school diploma, university degree); the mother’s marital status (married/cohabiting or unmarried/separated/divorced/widow); and the family’s disposable income. This last item was measured with the question “How do you make ends meet with your finances?” (very easily, quite easily or with some/great difficulty).

The mother’s health consciousness, defined as the degree to which individuals care about their health, was measured using a score specifically developed by Dutta-Bergman [18] and comprising five items rated on a scale of 1 to 5, where 1 = “strongly disagree” and 5 = “strongly agree”. The overall scores were then divided between three distribution tertiles, with the first tertile corresponding to the lowest level of health consciousness and the third corresponding to the highest one.

Finally, the mother’s health literacy was tested using the Italian version of the Newest Vital Sign (NVS) assessed for validity in a previous study [19], and classified on two levels: adequate (NVS score 4 or 5) or marginal/limited (NVS scores 0 to 3).

### Statistical analyses

A preliminary bivariate analysis was run to identify the distributions of EUE and EOE by socio-demographic, behavioral, and lifestyle variables. In particular: the χ2 test was applied to find differences in how the categorical variables were distributed by EUE and EOE; Student’s t-test was used to check for differences in the means of the continuous variables by EUE and EOE groups; and the Mann Whitney test was used for the discrete variables deriving from the SDQ scores.

Finally, a multivariable logistic regression was performed to test the association between EUE or EOE (dependent variable: third tertile as outcome) and the other variables investigated relating to socio-demographics, behavioral traits, and adherence to Mediterranean diet (as independent variables).

The STATA software (ver. 14) was used for all the statistical analyses.

### Ethics approval and consent to participate

This study was approved by the Ethical Committee at Padova Teaching Hospital. The children’s participation in the study was subject to the consent of the directors at the schools involved. Parents of all the children ultimately participating in the study then signed an informed consent form. All procedures complied with the ethical standards adopted by Padova Teaching Hospital, the Italian National Research Committee, and the 1964 Helsinki Declaration and subsequent revisions thereof, or comparable ethical standards. All the methods/procedures were performed in accordance with the relevant guidelines and regulations.

## Results

Table 1 shows the study sample’s characteristics, for both the children and their mothers. The proportion of males and females in the sample was similar, slightly more than half (54.5%) of the children being male, and most of the children (73.0%) were 8 years old. The children’s body mass index (BMI) was divided into two categories, with 71.3% of the sample underweight or normal weight, and 28.1% overweight or obese. Slightly more than one in five of the children involved in the study had a good adherence to the MD (20.8%).

**Table 1:** Characteristics of the study sample. Numbers (n) and percentages (%)

	Participants
**Variables concerning the child**	
Sex, *n (%)*	
Males	97 (54.5%)
Females	81 (45.5%)
Age, *n (%)*	
8 years old	130 (73.0%)
9 years old	48 (27.0%)
BMI, *n (%)*	
Underweight/normal-weight	127 (71.3%)
Overweight/obese	50 (28.1%)
Siblings, *n (%)*	
None	50 (28.1%)
1 or more	128 (71.9%)
Adherence to the MD, *n (%)*	
Good	37 (20.8%)
Poor-to-moderate	126 (70.8%)
Prosocial behavior, *median (range)*	8 (2–10)
Emotional symptoms, *median (range)*	1 (0–8)
Hyperactivity, *median (range)*	3 (0–10)
Peer problems, *median (range)*	1 (0–7)
Conduct problems, *median (range)*	2 (0–7)
Hours of sleep per day*, mean ± SD*	9.10 ± 0.65
**Variables concerning the mother**	
Health consciousness*, median (range)*	21 (4–25)
Health literacy, *n (%)*	
Adequately	69 (38.8%)
Marginal/limited	108 (61.2%)
Citizenship, *n (%)*	
Italian	154 (86.5%)
Other	23 (12.9%)
Marital status, *n (%)*	
Married/cohabiting	160 (89.9%)
Single/separated/divorced/widow	18 (10.1%)
Education level, *n (%)*	
Middle school or less	28 (15.7%)
High-school diploma	78 (43.8%)
University degree	72 (40.5%)
Disposable income, *n (%)*	
I make ends meet with some/great difficulty	56 (31.5%)
I make ends meet quite easily	86 (48.3%)
I make ends meet very easily	35 (19.7%)

Table 2 shows the distribution of the children’s emotional eating behavior by categorical variables, and particularly their demographic characteristics, BMI, adherence to the MD, and mother-related variables.

**Table 2:** Bivariate analysis between emotional eating (EUE and EOE) and categorical covariates

Risk factors	Emotional undereating (Yes 3rd tertile) ***N*** = 52	***p***-value χ2 test	Emotional overeating (Yes 3rd tertile) ***N*** = 56	***p***-value χ2 test
Sex*, % (n)*				
Male	25.8% (25)	0.30	28.9% (28)	0.62
Female	33.3% (27)		34.6% (28)	
Age*, % (n)*				
8 years old	30.0% (39)	0.77	26.2% (34)	**0.05**
9 years old	27.1% (13)		45.8% (22)	
BMI*, % (n)*				
Underweight/normal-weight	32.3% (41)	0.05	29.9% (38)	0.90
Overweight/obese	20.0% (10)		34.0% (17)	
Siblings*, % (n)*				
None	26.0% (13)	0.61	30.0% (15)	0.86
1 or more	30.5% (39)		32.0% (41)	
Adherence to the MD*, % (n)*				
Poor-to-moderate	34.1% (43)	0.06	34.1% (43)	0.08
Good	16.2% (6)		21.6% (8)	
Health consciousness*, % (n)*				
First and second tertile	29.6% (37)	1	32.0% (40)	1
Third tertile (high)	28.3% (15)		30.2% (16)	
Citizenship*, % (n)*				
Italian	29.2% (45)	0.30	34.4% (53)	**0.02**
Other	26.1% (6)		8.7% (2)	
Marital status*, % (n)*				
Married/cohabiting	30.0% (48)	0.71	29.4% (47)	**0.02**
Single/separated/divorced/widow	22.2% (4)		50.0% (9)	
Education level*, % (n)*				
Middle school or less	14.3% (4)	0.22	21.4% (6)	0.30
High-school diploma	33.3% (26)		37.2% (29)	
University degree	30.6% (22)		29.2% (21)	
Disposable income*, % (n)*				
Some/great difficulty	32.1% (18)	0.91	33.3% (18)	0.89
Quite easily	28.8% (23)		35.0% (28)	
Very easily	29.4% (10)		30.3% (10)	

Percentages (%), numbers (n), *p*-values

Table 3 shows the results of the bivariate analysis between emotional eating and the discrete variables deriving from the SDQ scores, by EUE and EOE.

**Table 3:** Bivariate analysis of relations between emotional eating (EUE and EOE) and discrete and continuous variables

Risk factors	Emotional undereating (No 1st and 2nd tertiles)	Emotional undereating (Yes 3rd tertile)	***p***-value	Emotional overeating (No 1st and 2nd tertiles)	Emotional overeating (Yes 3rd tertile)	***p***-value
Prosocial behavior, *median (range)*	8 (2–10)	8 (4–10)	0.51	8 (2–10)	8 (4–10)	0.92^
Emotional symptoms, *median (range)*	1 (0–8)	2 (0–6)	**< 0.01**	1 (0–6)	2 (0–8)	**< 0.01^**
Hyperactivity, *median (range)*	3 (0–10)	3 (0–9)	0.55	2 (0–9)	4 (0–10)	**< 0.01^**
Peer problems, *median (range)*	1 (0–6)	1 (0–7)	0.32	1 (0–7)	1 (0–6)	0.40^
Conduct problems, *median (range)*	2 (0–6)	2 (0–7)	0.43	1 (0–7)	2 (0–5)	**0.03^**
Hours of sleep per day*, mean ± SD*	9.07 ± 0.06	9.15 ± 0.07	0.43	9.10 ± 0.07	9.06 ± 0.08	0.70*

Medians and ranges for discrete variables, means ± SD for continuous variables, *p*-values

* Student’s t-test

^ Mann Whitney test

Table 4 shows the results of the logistic regression models of the association between the children’s emotional eating behaviors (EUE and EOE) and their socio-demographics, behavioral traits, and dietary habits. Concerning the children’s behavioral traits, multivariate analysis revealed that EUE was positively associated with emotional symptoms (OR 1.72; 95% CI 1.11–2.67), while EOE was positively associated with both emotional symptoms (OR 2.01; 95% CI 1.29–3.13) and hyperactivity (OR 2.80; 95% CI 1.59–4.92), and inversely associated with peer problems (OR 0.50; 95% CI 0.25–0.99). EUE was positively associated with the number of siblings (OR 1.50; 95% CI 1.03–2.18), and inversely associated with a good adherence to the MD (OR 0.25; 95% CI 0.08–0.84).

**Table 4:** Multivariable logistic regressions between outcome variables (EUE and EOE) and covariates

	Emotional undereating (Yes 3rd tertile)^**a**^				Emotional overeating (Yes 3rd tertile)^**b**^			
	OR	95% CI		***p***-value	OR	95% CI		***p***-value
		Lower limit	Upper limit			Lower limit	Upper limit	
Sex *(ref: female)*	1.03	0.45	2.34	0.95	1.03	0.43	2.43	0.95
BMI *(ref: underweight/normal weight)*	0.45	0.17	1.17	0.10	1.27	0.47	3.44	0.64
Number of siblings	**1.50**	**1.03**	**2.18**	**0.04**	0.98	0.70	1.37	0.92
Adherence to the MD *(ref: poor-to-moderate)*	**0.25**	**0.08**	**0.84**	**0.02**	0.58	0.20	1.69	0.32
Prosocial behavior *(ref: first and second tertiles)*	1.35	0.89	2.06	0.16	0.80	0.50	1.26	0.33
Emotional symptoms *(ref: first and second tertiles)*	**1.72**	**1.11**	**2.67**	**0.02**	**2.01**	**1.29**	**3.13**	**< 0.01**
Hyperactivity *(ref: first and second tertiles)*	0.74	0.45	1.22	0.24	**2.80**	**1.59**	**4.92**	**< 0.01**
Peer problems *(ref: first and second tertiles)*	1.05	0.58	1.91	0.86	**0.50**	**0.25**	**0.99**	**0.047**
Conduct problems *(ref: first and second tertiles)*	1.42	0.87	2.31	0.16	0.73	0.43	1.24	0.25
Health consciousness *(ref: first and second tertiles)*	1.51	0.52	4.35	0.45	1.43	0.48	4.26	0.52
Citizenship *(ref: Italian)*	0.73	0.18	2.98	0.66	0.19	0.03	1.16	0.07
Marital status *(ref: married/cohabiting)*	0.43	0.08	2.32	0.33	3.18	0.65	15.59	0.15
Hours of sleep per day	1.06	0.53	2.14	0.87	0.98	0.49	1.97	0.96
Mother’s education								
Middle school or less	1.00				1.00			
High-school diploma	3.03	0.67	13.78	0.15	1.43	0.37	5.51	0.60
University degree	4.30	0.90	20.52	0.07	0.73	0.18	2.97	0.66
Disposable income								
I make ends meet with some/great difficulty	1.00				1.00			
I make ends meet quite easily	0.72	0.27	1.91	0.51	1.11	0.40	3.07	0.84
I make ends meet very easily	0.43	0.11	1.68	0.22	0.51	0.12	2.14	0.36

Odds ratios (OR), 95% confidence intervals (95% CI), and *p*-values

in bold *p* < 0.05

^**a**^Pseudo R^2^ = 0.16, ^**b**^Pseudo R^2^ = 0.24

## Discussion

This study examined the associations between emotional eating and behavioral traits in a sample of 8- to 9-year-old Italian primary-school children. It found EUE positively associated with emotional symptoms, and EOE positively associated with emotional symptoms and hyperactivity, and inversely associated with peer problems. EUE also showed a positive association with a social environment variable (number of siblings) and an inverse association with a good adherence to the MD.

To the best of our knowledge, the present study is the first to have found a negative association between EUE and adherence to the MD in childhood. In particular, a good adherence to the MD was associated with a lower risk of EUE, while no such association emerged for EOE. Conversely, a previous study of Jalo et al. found a direct association between emotional overeating and an unhealthy diet in a large international sample of 9- to 11-year-old children [9]. Other studies conducted on adults and on 12- to 15-year-olds showed that emotional eating is associated with the consumption of sweet and high-fat foods [20–22]. On the other hand, there have also been reports of no association between emotional eating and the consumption of snacks, sweet foods, or fatty foods in children aged from 5 to 12 years [23, 24]. Models based on psychodynamic and developmental perspectives generally suggest that eating disorders and their various symptoms might be considered as an impaired cognitive capacity to process and regulate emotions [25]. The challenge for caregivers is to provide structure and boundaries without limiting children’s eating autonomy to such an extent that they no longer self-regulate their eating, seeing external factors as eating cues instead [26]. Feeding styles capture the overall emotional climate of meals and are measured along two dimensions: responsiveness (represented by warmth, acceptance, and involvement during feeding) and demandingness (represented by parental control and supervision of feeding). Using these two dimensions, feeding behavior is often classified in one of four ways, as authoritarian, authoritative, indulgent or uninvolved [27]. Previous studies found the indulgent feeding style associated with children being less able to self-regulate their eating, emphasizing the importance of setting boundaries around food [27, 28]. In this view, a good adherence to the MD could be part of a broadly positive family approach to food. It may be that children with a healthy diet orient their negative emotions towards different coping strategies, avoiding the risk of EUE.

The present study also found evidence of behavioral traits being involved in both EUE and EOE, the Emotional Symptoms subscale of the SDQ being associated with both emotional eating behaviors. The subscale includes questions about frequency of headaches, stomach-ache or sickness, children’s worries, fears, unhappy feelings, and how they feel about new situations [13]. Food in general (and especially palatable food) can boost our mood [29], and eating can reduce the intensity of negative emotions [30]. Children whose parents offer food as an emotional regulation strategy may be prone to overeating and could learn to associate food with pleasure, leading to a greater reliance on food as a way of coping with emotions, instead of eating to meet their nutritional needs [31]. Psychological theories to explain emotional undereating are virtually non-existent, however, and EUE has been attributed to biological mechanisms [30]. In actual fact, the most natural response to emotional distress is the suppression of hunger due to a decreased gut activity in the presence of emotional arousal [32], sympathetic activation and glucocorticoid release [33]. These former psychological and latter biological mechanisms can partially disentangle our findings.

Moreover, we found relationship between EOE and other two SDQ subscales, hyperactivity and peer problems. The Hyperactivity subscale of the SDQ [13] defines hyperactivity as a tendency to be restless, overactive, constantly fidgeting or squirming, easily distracted, and with a poor concentration/attention span; the peer problems construct concerns a child’s tendency to play alone, and to get on better with adults than with other children [34]. These behavioral traits are considered a stress factor [24]. The association between stress (including peer problems and hyperactivity) and EOE is generally accepted, as it is defined as overeating as a reaction to emotional arousal [24]. Eating may be used as a way to cope with stress, by lowering stress levels and increasing feelings of reward [35, 36].

More broadly, a study investigating the association between core symptoms of attention deficit hyperactivity disorder (ADHD) and disordered eating found that the inattentive symptoms of ADHD were associated with decreased awareness of internal signals of hunger/satiety, and this deficit was positively associated with disordered eating, particularly binge/disinhibited eating [37]. Our data partially confirmed the existing literature: in fact, EOE is positively associated with hyperactivity while peer problems are negatively associated with EOE in our results, in contrast with the literature to date. Given our conflicting data, other studies will be necessary to confirm and shed light on this finding.

Finally, we found an association between number of siblings and EUE: for each additional sibling, the odds of a child showing EUE behavior rose by 50%. When investigating emotional eating among adolescents, De Leeuw et al. found that siblings who are close, showing affection and empathy, tend to be more alike in their emotional eating behavior [38]. Further research will be needed, also investigating the siblings’ characteristics, to elucidate why the number of siblings might affect children’s emotional eating – and, more broadly, how the family setting could influence this behavior.

We found no association between BMI and emotional eating, in line with other studies [9, 23, 39], although some papers have supported this association. Consistently with our results, Jalo et al. emphasized their somewhat controversial finding that emotional eating was associated with the healthiness of people’s diet, but not with their BMI. It should be noted that dietary patterns are scored in terms of nutritional quality, not energy intake, while the latter may be more closely related to BMI [9]. When Jalo et al. discussed their results, they made the point that most of the literature reporting positive associations between emotional eating and BMI (being overweight) considered parent-reported emotional eating, while studies investigating child-reported emotional eating found mostly inverse associations [9]. These discrepancies could go to show the limitations of using self-reported data, which might be influenced by social desirability. The use a self-reported measure of diet and BMI is a shortcoming of our study too. There are also other limitations that need to be considered when it comes to interpreting the results of the present research. First, the design of this study did not enable us to establish any causality for the significant associations examined. It would be well worth seeking such possible causal relationships and mechanisms, using appropriate study designs. Our data on emotional eating and other variables were obtained by means of questionnaires. This method is not objective and may suffer from a social desirability bias, though we can assume that this potential source of bias was contained by our use of anonymous questionnaires. Nonetheless, mothers can be sensitive about their children’s behavior, so our findings may be biased by their having exaggerated or played down their children’s eating habits. They might also have failed to answer the questions correctly because they do not know what their children behavior away from home (e.g. at school). Another shortcoming could be the relatively low participation rates, which might determine a selection bias if the sample that took part in the study is not proportional across subgroups of the exposure and outcome variables in the source population. The low participation rates and consequently small sample size also affects the accuracy of the study’s estimates.

Nevertheless, the study has the strength, given the paucity of literature, to address a relevant issue, providing an overall picture of the risk factors of emotional eating in children, considering also the particular period of pandemic that affected the psychological wellbeing of children [40].

## Conclusions

In conclusion, our study identified associations between emotional eating and children’s dietary patterns and some behavioral traits (emotional symptoms, hyperactivity and peer problems). It could be useful to improve parents’ awareness so that they can anticipate and pay more attention to the problem of emotional eating, and help their children to work on their stress coping skills (such as taking a problem-solving approach or asking for help) instead of seeking solace in food [24]. Adherence to the MD should be reinforced as well, by means of health promotion schemes at school, for example.

## Data Availability

The datasets analyzed during the current study are not publicly available but are available from the corresponding author on reasonable request.

## Abbreviations

EOE: Emotional overeating
EUE: Emotional undereating
MD: Mediterranean diet
CEBQ: Children’s Eating Behavior Questionnaire
SDQ: Strengths and Difficulties Questionnaire
NVS: Newest Vital Sign
BMI: Body mass index
OR: Odds ratio
CI: Confidence interval
ADHD: Attention deficit hyperactivity disorder
